# Autonomic stress response of physiotherapy student in the different scenarios of an objective structured clinical examination

**DOI:** 10.1186/s12909-022-03903-9

**Published:** 2022-11-24

**Authors:** Beatriz Martínez-Pascual, Ana Ramírez-Adrados, Silvia Fernández-Martínez, Cristina Gonzalez-de-Ramos, Valentín E. Fernández-Elías, Vicente J. Clemente-Suárez

**Affiliations:** Faculty of Sport Sciences, Universidad Europea de Madrid, Tajo street, s/n, Madrid 28670 Villaviciosa de Odón, Spain

**Keywords:** Autonomic stress response- complexity- heart rate variability -objective structured clinical evaluation- physiotherapy- students

## Abstract

The aim of the present research was to analyse modifications in the autonomic stress response of Physiotherapy students undergoing a 12-scenario Objective Structured Clinical Evaluation (OSCE). A total of 86 last year students of the Physiotherapy bachelor’s degree (27.29 years (SD = 6.66); 36 females and 50 males) randomly assigned were monitored during the complete OSCE to measure heart rate variability (HRV) in temporal, frequency, and non-linear domains. The HRV analysed showed a large anticipatory stress response of students maintained during the entire evaluation. The stress response varied regarding OSCE station complexity and demands and the highest sympathetic response was not found in higher emotional scenarios.

The autonomic modulation monitoring allows teachers to design OSCE scenarios more adapted to the students, limiting the effect of the stress response to allow a better performance.

## Introduction

Since its development in 1970 [1], the Objective Structured Clinical Evaluation (OSCE) is worldwide considered as one of the most rational, effective and dependable methods to assess clinical performance while increasing the educational impact [2–5]. In health care area, assessment tools must be able to evaluate the combination of knowledge, skills and behaviours influencing clinical performance [6] . Conventional assessment methodologies for clinical competences often lacks structure and standardization, present poor inter-rater reliability and not minimize examiner bias. Therefore, the same student can obtain diverse results when being evaluated of the same competence by different evaluators [7]. Over the past few decades, the OSCE has been proven to be a reliable and valid tool that can be used to assess all three learning domains (cognitive, affective and psychomotor), the “show how” and “does how” described in the Millar pyramid [8, 9]. It has been widely adopted as the examination for clinical performance in several health disciplines in undergraduate and postgraduate [10].

Previous research [11] has shown as uncertain and unpredictable environments produce a large anxiogenic response in students that could negatively affect their ability to cope with the acquisition of learning process [11–13]. Evaluation context as final degree defence, clinical stays or practical evaluations produce a large stress response of students due to this uncertainty and unpredictability [14–18].

Recent studies have observed this anticipatory anxiety response in psychology students during the OSCE evaluation [13]. Nursing students show a lack of habituation response while practising in real context or doing their OSCE as well as chemistry students in their laboratory practices [16]. The increased stress response was also observed in physiotherapy students, where a high anticipatory anxiety response was evaluated at both, subjective and objective level, in clinical simulations, not achieving the expected habituation [13]. In this line, several studies based on students self-perception questionnaires, have reported the OSCE as an “anxiety provoking assessment method” due to scenarios timing, the proximity of the evaluator and the high expectation to succeed [7, 19].

Academic events such as OSCE, practices and final degree defence confront the students with a potentially stressful and warning situation. When the organism interprets a stimulus as threatening, the sympathetic autonomous nervous system and the hypothalamic-pituitary-adrenal axis are activated, increasing production of cortisol, adrenaline, and noradrenaline [20, 21]. Even through these actions are not conscious, they would induce both, structural and functional changes in neurons, producing a general decrease in the executive function’s performance [22]. In this line, previous research used the monitorization of the autonomic modulation by the heart rate variability (HRV) to analyse the autonomic stress response. These new methodology was applied in eliciting environments as sport, military, health and education areas [23–26]. Specifically, there are not much research on physiotherapy OSCE, despite the importance it has in the formation of these students. Then, the aim of the present study was to analyse modifications in the autonomic stress response of Physiotherapy last year students in a 12-scenarios OSCE. The starting hypothesis were: (i) autonomic stress response would vary according to the demands of each OSCE scenario and (ii) students would present a higher sympathetic response in the scenarios that had a higher emotional impact.

## Material and methods

### Participants

Every student enrolled in the final year of Physiotherapy degree take the OSCE evaluation as a compulsory part of a course called Practicum. Therefore, participants were a convenience sample composed by a total of 86 students (42 women and 58 men). Medium age was 27.29 (SD = 6.66). Every student had the same experience prior to facing the OSCE evaluation so they all shared same starting point regarding competences acquisition during the degree. The procedure was conducted following the Helsinki Declaration (as revised in Brazil, 2013), all the participants filled an informed consent form before starting the research and all the procedures were approved by the University Ethic Committee.

### Measurements and instruments

Heart rate variability was registered to evaluate the autonomic stress response of the students using a validated [27] Polar V800 heart rate monitor (Polar, Kempele, Finland). Subsequently, the R-R intervals were examined with the Kubios HRV software (v3.0, Biosignal Analysis and Medical Imaging Group, University of Kuopio, Finland). We assessed HRV through the time-domain, frequency-domain and nonlinear spectrum variables.

Time-domain variables

- HRmean: mean heart rate
- RMSSD: the square root of the average of the sum of the differences squared between normal adjacent R-R intervals
- PNN50: the percentage of differences between normal adjacent R-R intervals greater than 50 ms

Frequency-domain variables:

- LF: the low-frequency band in normalized units
- HF: the high-frequency band in normalized units
- LF/HF: the ratio between low and high frequency band.

Nonlinear spectrum variables:

- SD1: the sensitivity of the short-term variability
- SD2: the sensitivity of the long-term variability

### Procedure

It is an observational study to analyse the autonomic response in physiotherapy students during their OSCE. The evaluation was conducted in the simulated hospital located in the Faculty of Health Sciences of the University. The OSCE was composed by 12 scenarios and the OSCE coordinator randomly assigned every student, according to their last name alphabetical order, to the scenario they must start in and the subsequent order of scenarios.

The process begins with the placement of the Polar V800 heart monitor (Polar, Kempele, Finland) before starting the evaluation. Students had 10 min to solve the unknown situation with all the knowledge and competences acquired during the degree. Scenarios were separated by 2 min. All participants received the same written information for 2 min prior to start each scenario.

The OSCE analysed was composed by the following scenarios (Scenario 1 is named as S1 and so are the others): S1: identifying red flags in a simulated patient consultation; S2: conducting physical examination of a simulated patient; S3: modifying the treatment of a simulated patient according his evolution; S4: preparing a clinical reasoning (non-interactive written scenario); S5: practising a cardiopulmonary resuscitation (CPR); S6: break: free time; S7: attending a respiratory simulated patient in Intensive Care Unit (ICU); S8: mobilization: carrying out posture changes of a neurological simulated patient.; S9: writing a patient physiotherapy report (non-interactive written scenario); S10: attending a professional simulated sportsmen/women in a consultation; S11: interacting with an urogynaecology simulated patient and her companion; S12: break: free time.

### Statistical analysis

We carried out all statistical analyses using the IBM statistical Package for the Social Sciences (SPSS) software version 24.0 for Windows. Data in the present study are presented as mean ± standard deviation (SD). Kolgomorov-Smirnov test revealed that all variables were normally distributes. Subsequently, data were analyzed by one-way analysis of variance with repeated measures (ANOVA). After a significant F-value (Greenhouse–Geisser adjustment for sphericity), pairwise differences between means were identified by using Bonferroni post-hoc procedure. Effect size was estimated by calculating partial eta squared (η^2^p). The level of significance for all the comparisons was set at *p* ≤ 0.05.

## Results

In the HRV time-domain variables, the one-way repeated measures ANOVA showed significant differences in HRmean, RMSSD and PNN50 (Table 1) among the different OSCE scenarios. Post hoc analysis showed that HRmean was significantly greater in S1 compared to S4 (*p* < 0.001), S5 (*p* = 0.004), S6 (*p* < 0.001), S7 (*p* < 0.001), S8 (*p* < 0.001), S9 (*p* < 0.001), S10 (*p* < 0.001), S11 (*p* < 0.001), and S12 (*p* < 0.001). In S3 compared to S6 (*p* = 0.003), S8 (*p* = 0.002), S8 (*p* < 0.001), S9 (*p* < 0.001), S10 (*p* < 0.001), S11 (*p* < 0.001), and S12 (*p* < 0.001). In S3 compared to S6 (*p* = 0.006), S8 (*p* = 0.004), S9 (*p* < 0.001), S10 (*p* < 0.001), S11 (*p* < 0.001), and S12 (*p* < 0.001). In S4 compared to S9 (*p* = 0.001), S10 (*p* = 0.007), S11 (*p* = 0.006), and S12 (*p* = 0.006). And in S5 compared to S8 (*p* = 0.042), S9 (*p* < 0.001), S11 (*p* = 0.003), and S12 (*p* = 0.008).

**Table 1:** Time-domain heart rate variability results

	HRmean (bpm)	RMSSD (ms)	PNN50 (%)
**Scenario 1**	104.63 (20.13)	27.89 (17.76)	7.13 (8.73)
**Scenario 2**	101.33 (19.97)	30.59 (23.57)	8.82 (11.84)
**Scenario 3**	99.79 (19.43)	31.88 (23.33)	9.24 (10.72)
**Scenario 4**	97.55^a^ (19.45)	31.99 (27.60)	9.39 (10.84)
**Scenario 5**	96.99^a^ (18.97)	34.09 (27.79)	10.33^a^ (12.05)
**Scenario 6**	94.89^abc^ (18.76)	34.75 (28.05)	10.67^a^ (11.45)
**Scenario 7**	94.75^ab^ (17.13)	33.91 (27.82)	10.40^a^ (11.89)
**Scenario 8**	93.00^abce^ (16.78)	35.68^a^ (26.15)	11.93^ab^ (13.47)
**Scenario 9**	91.43^abcde^ (15.84)	36.37^abd^ (27.96)	11.74^a^ (11.94)
**Scenario 10**	92.09^abcd^ (16.87)	36.68^abcd^ (24.48)	12.44^acd^ (12.11)
**Scenario 11**	91.37^abcde^ (17.96)	39.68^abcde^ (29.76)	13.92^abcde^ (14.23)
**Scenario 12**	91.40^abcde^ (16.89)	38.30^abcd^ (23.29)	12.86^abcd^ (12.56)
**F**	21.156	9.644	10.595
**P**	0.000	0.000	0.000
**η**^**2**^**p**	0.205	0.105	0.114

^a^Significantly different than S1

^b^Significantly different than S2

^c^Significantly different than S3

^d^Significantly different than S4

^e^Significantly different than S5

^f^Significantly different than S6

^g^Significantly different than S7

Regarding RMSSD, post hoc analysis showed that RMSSD was significantly lower in S1 compared to S8 (*p* = 0.002), S9 (*p* = 0.002), S10 (*p* < 0.001), S11 (*p* < 0.001), and S12 (*p* < 0.001). In S2 compared to S9 (*p* = 0.014), S10 (*p* = 0.027), S11 (*p* = 0.004), and S12 (*p* = 0.001). In S3 compared to S10 (*p* = 0.022), S11 (*p* = 0.003), and S12 (*p* = 0.004). In S4 compared to S9 (*p* = 0.039), S10 (*p* = 0.045), S11 (*p* = 0.001), and S12 (*p* = 0.016). And in S5 compared to S11 (*p* = 0.020).

Finally, PNN50 post hoc analysis showed that PNN50 was significantly inferior S1 compared to S5 (*p* = 0.045), S6 (*p* = 0.018), S7 (*p* = 0.001), S8 (*p* < 0.001), S9 (*p* < 0.001), S10 (*p* < 0.001), S11 (*p* < 0.001), and S12 (*p* < 0.001). In S2 compared to S8 (*p* = 0.028), S11 (*p* = 0.004), and S12 (*p* = 0.004). In S3 compared to S10 (*p* = 0.007), S11 (*p* = 0.001), and S12 (*p* = 0.004). In S4 compared to S10 (*p* = 0.008), S11 (*p* = 0.001), and S12 (*p* = 0.002). And in S5 compared to S11 (*p* = 0.001).

Similarly, the nonlinear spectrum of HRV also showed significant differences in SD1 and in SD2 between several OSCE scenarios (Table 2). Post hoc analysis showed that SD1 was significantly lower in S1 compared to S8 (*p* = 0.002), S9 (*p* = 0.002), S10 (*p* < 0.001), S11 (*p* < 0.001), and S12 (*p* < 0.001). In S3 compared to S10 (*p* = 0.022), S11 (*p* = 0.003), and S12 (*p* = 0.004). In S4 compared to S9 (*p* = 0.039), S10 (*p* = 0.045), S11 (*p* = 0.001), and S12 (*p* = 0.016). And in S5 compared to S11 (*p* = 0.019). Further, post hoc analysis indicated that SD2 was significantly smaller in S1 compared to S3 (*p* = 0.036), S4 (*p* = 0.011), S5 (*p* < 0.001), S6 (*p* < 0.001), S7 (*p* < 0.001), S8 (*p* < 0.001), S9 (*p* < 0.001), S10 (*p* < 0.001), S11 (*p* < 0.001) and S12 (*p* < 0.001). In S2 compared to S4 (*p* = 0.035), S5 (*p* = 0.002), S6 (*p* < 0.001), S7 (*p* < 0.001), S8 (*p* < 0.001), S9 (*p* < 0.001), S10 (*p* < 0.001), S11 (*p* < 0.001) and S12 (*p* < 0.001). In S3 compared to S7 (*p* = 0.006), S8 (*p* = 0.040), S9 (*p* < 0.001), S10 (*p* < 0.001), S11 (*p* < 0.001), and S12 (*p* < 0.001). In S4 compared to S9 (*p* < 0.001), S10 (*p* = 0.001), S11 (*p* < 0.001) and S12 (*p* = 0.001). In S5 compared to S9 (*p* = 0.004), S10 (*p* = 0.003), S11 (*p* < 0.001) and S12 (*p* = 0.001). And in S8 compared to S11 (*p* = 0.037).

**Table 2:** Nonlinear heart rate variability results

	SD1 (ms)	SD2 (ms)
**Scenario 1**	19.73 (12.56)	51.58 (19.67)
**Scenario 2**	22.73 (12.14)	54.00 (21.61)
**Scenario 3**	22.55 (16.50)	57.03^a^ (24.12)
**Scenario 4**	22.63 (19.52)	59.13^ab^ (23.82)
**Scenario 5**	24.12 (19.66)	60.48^ab^ (23.29)
**Scenario 6**	24.58 (19.84)	62.97^ab^ (24.43)
**Scenario 7**	23.99 (19.68)	63.54^abc^ (23.82)
**Scenario 8**	25.24^a^ (18.50)	63.08^abc^ (21.64)
**Scenario 9**	25.73^ad^ (19.78)	66.52^abcde^ (24.32)
**Scenario 10**	25.94^acd^ (17.32)	67.16^abcde^ (22.56)
**Scenario 11**	28.07^acde^ (21.05)	68.85^abcdeh^ (24.70)
**Scenario 12**	27.10^acd^ (16.48)	67.95^abcde^ (22.23)
**F**	7.643	22.801
**P**	0.000	0.000
**η**^**2**^**p**	0.085	0.218

^a^Significantly different than S1

^b^Significantly different than S2

^c^Significantly different than S3

^d^Significantly different than S4

^e^Significantly different than S5

^h^Significantly different than S8

However, the ANOVA analysis of the HRV frequency-domain did not show significant differences in LF HF and LF/HF ratio variables (Table 3).

**Table 3:** Frequency-domain heart rate variability results

	LF/HF (n.u.)	LF (n.u.)	HF (n.u.)
Scenario 1	4.07 (2.66)	75.21 (11.45)	24.72 (11.40)
Scenario 2	4.63 (3.01)	77.18 (12.18)	22.74 (12.14)
Scenario 3	4.55 (2.69)	76.95 (12.51)	22.97 (12.45)
Scenario 4	4.57 (2.57)	77.81 (11.19)	22.12 (11.16)
Scenario 5	4.61 (2.74)	77.65 (12.11)	22.27 (12.05)
Scenario 6	4.46 (2.62)	77.41 (11.51)	22.51 (11.46)
Scenario 7	4.83 (2.81)	78.49 (11.03)	21.44 (10.99)
Scenario 8	4.34 (2.74)	76.93 (11.55)	23.00 (11.50)
Scenario 9	4.43 (2.71)	77.46 (10.72)	22.48 (10.69)
Scenario 10	4.50 (2.61)	77.66 (11.13)	22.27 (11.09)
Scenario 11	4.49 (3.02)	76.90 (11.82)	23.03 (11.78)
Scenario 12	4.30 (2.35)	77.12 (10.80)	22.81 (10.76)
F	1.475	1.396	1.398
P	0.135	0.169	0.168
η^2^p	0.018	0.017	0.017

## Discussion

The aim of this study was to analyse modification in the autonomic stress response of last year physiotherapy students in an OSCE composed of 12-scenario randomly assigned to students. The first hypothesis was confirmed because stress response varied regarding OSCE station complexity and demands. The second hypothesis was not fulfilled since higher sympathetic response was not found in higher emotional scenarios.

We found a large anticipatory stress response of students as the low values in PNN50, RMSSD, SD1, SD2, HF and high LF values showed. This result was related to an increased sympathetic nervous system modulation due to the OSCE was perceived as unknown, uncertain, uncontrollable, and even threatening for students. Then, the OSCE produced an increase in the sympathetic modulation that mobilize organic resources to prepare students to face the novel and uncontrollable situations of the OSCE. In the present study the OSCE is a mandatory event to their academic grades, what can act as a stressor for students. Moreover, it was the first OSCE experience for the students. The same autonomic response has been previously studied in other educational context, as final degree dissertation or clinical stays and in stressful environments like military combat training, parachute jumps or extreme sport events as ultra-endurance races [28–31]. In these eliciting environments an increased sympathetic response was also evaluated, highlighting the universal stress response of human body to face threats, independently of the nature of the stimuli [14, 32, 33].

As the three HRV domains used showed, a maintained sympathetic modulation was observed during the whole OSCE. The no reduction in the anxiogenic response could be explained because students had no experience in this type of clinical evaluation which is a novel, incontrollable and unpredictable environment for them. On this point, it is interesting to remark that the analysed OSCE had been only used once, in the previous academic year. There have not been new stations added or modified scenarios. Moreover, in contrast with other OSCE in physiotherapy in the world, in our study students face a higher number of stations and taps in every aspect of practice (musculoskeletal, respiratory, neuromuscular and obstetrics) [34]. This lack of habituation response was also evaluated in other challenging education contexts as nursing students during an OSCE, pharmacy students in real chemistry laboratory practices and physiotherapy students in the defence of their final degree dissertation [15, 16, 35].

Although this sympathetic modulation is maintained, the HRV analysed showed a different autonomic stress response depending on the nature of the scenario and regardless the order of appearance in the OSCE. In our initial hypothesis students would present a higher sympathetic response in the scenarios that had a higher emotional impact: S7 (ICU patient), S8 (neurological patient) and S11 (urogynaecology patient with a companion). Contrary to our hypothesis results showed how the most stressful scenarios were S1 (red flags), S2 (physical examination) and S3 (modifying the treatment). In this regard, it is important to highlight that the sequence of scenarios is different for each student. The three of them have in common that students face simulated patients (actors), in contexts deeply linked to the basic underpinning physiotherapy profession in which the clinical reasoning and decision-making skills are essential. This could explain why the ICU station (S7) initially considered as high emotional impact scenario presented a lower sympathetic modulation as it uses a mannequin instead of an actor, what could decrease authenticity of setting and context [7]. Even though in S8 and S11 students face patients, these scenarios demand communication and treatment skills over the clinical reasoning or decision making. This difference in difficulty and stress perception between students and teachers facing an OSCE evaluation have already been studied in previous studies [18].

The S1, the red flags station, was the most stressful situation evaluated in the OSCE. Specifically, PNN50 presented significantly lower values in S1, compared with S5, S6, S7, S8, S9, S10, S11 and S12. This scenario requires from the student to detect red flags what means being vigilant to signs and symptoms that can be medical emergency or are suggestive of sinister pathology. It requires dexterity in clinical reasoning, one of the most difficult skills to develop in the Physiotherapy curriculum. By contrary, the lower sympathetic response was found in S11 as show high values in every HRV parameters, even higher than in the resting scenarios (break). It is a communication station in which students interview an urogynaecology patient with a companion. The fact of interacting with 2 actors do not increase the stress response as expected, what could be justified by the fact that urogynaecology is a minority physiotherapy specialty that could be considered less useful in their future profession [18]. Besides that, communication skills are trained in depth during the whole programme, through simulated scenarios and clinical practices what could also explain these results. The sympathetic modulation was maintained even in the resting scenarios, S6 and S11. As in previous research [13], the recovery scenarios were not enough to elicit a situation of calm and relaxation to decrease the anxiogenic response, increasing the sympathetic modulation and a new anticipatory anxiety response. Moreover, during these scenarios students sat waiting in a corridor surrounded by examiners what could increase the stress.

Interaction among examiners and students have been highlighted as stressor by previous studies [7, 19] using indirect tools (surveys) to examine stress. According to our HRV results, this could explain the high stress response in the non-interactive written scenarios (S4 and S9) in which students do not interact with patients but only with examiners. In addition, the demands in those scenarios requires higher level cognitive processes to access the information (analysing, evaluating, and creating) that could be negatively affected by the sympathetic modulation what could increase the anxiogenic response [36].

To summarize main findings, we can highlight that we found a large anticipatory stress response of undergraduate physiotherapy students and a maintained sympathetic modulation during the whole OSCE. Students undertook the OSCE for the first time what could explain the lack of habituation response.

The HRV analysed showed a different autonomic stress response depending on the nature and contents of the scenario and regardless their individual order of appearance in the OSCE:

The use of HRV to analyse autonomic stress response is a novel tool and it was applied to different stressful scenarios as sport, military, health and academics [22, 24, 25, 37, 38]. In this line, depending on the context and participants evaluated, different sensitivity to identify the autonomic response of the different HRV analysis domains were found. In the present research the HRV parameters sensitive to identify the autonomic modulation were related to the time domain (PNN50 and RMSSD) and nonlinear domain (SD1 and SD2). This result was in concordance with other studies conducted with physiotherapy students in their clinical practice [39]. However, in other research with psychology students, it was found how the sensitive HRV parameters to identify the autonomic modulation were PNN50, RMSSD, SD1, SD2, and HF [38]. In sport context, the sensitive parameters were the PNN50, RMSSD, and HF. It can be observed the impact on the context, the characteristics of the sample and the evaluation period on the HRV parameters´ sensitivity to identify autonomic modulation [23]. More research is needed to better understand the HRV modifications in different contexts and populations.

### Practical applications

The autonomic response is directly associated with variations in HRV parameters that are objectively measurable. The use of instruments to measure HRV in real time during OSCE could be a useful and valid tool to help teachers to understand and evaluate the complexity of the different scenarios designed in the OSCE evaluation. The stress response monitoring allows teachers to design OSCE scenarios more adapted to the students, limiting the effect of the stress response to allow a better performance. Patient interaction, authenticity of setting and professional contexts should be carefully monitored to vary the difficulty or complexity of stations. Students can benefit from the use of these instruments to obtain an objective measure of their stress level to use strategies to cope and reduce stress and improve cognitive functioning.

### Limitations and future research lines

The principal limitation of the present study was the non-control of stress hormones such as cortisol and alpha amylase. Future research might seek to address these issues. It would be also interesting to analyse other variables that could modulate the stress response of students such as the psychological profile to develop coping strategies for those less adaptative profiles [40, 41].

## Data Availability

The datasets generated and/or analysed during the current study are not publicly available due confindentiality agreements approved by the Universidad Europea Ethical Commitee, but are available from the corresponding author on reasonable request.
